# A survey of trainee specialists experiences at the University of Cape Town (UCT): Impacts of race and gender

**DOI:** 10.1186/1472-6920-9-26

**Published:** 2009-05-27

**Authors:** Leslie London, Sebastiana Kalula, Bonga Xaba

**Affiliations:** 1 Transformation and Equity Portfolio, Health Sciences Faculty and School of Public Health and Family Medicine, University of Cape Town, Cape Town, South Africa; 2 Department of Medicine and Postgraduate Support Portfolio, University of Cape Town, Cape Town, South Africa; 3 Transformation and Equity Portfolio, Health Sciences Faculty, University of Cape Town, Cape Town, South Africa

## Abstract

**Background:**

Efforts to redress racial and gender inequalities in the training of medical specialists has been a central part of a dedicated programme in the Faculty of Health Sciences at the University of Cape Town (UCT). This study aimed to describe trends in race and gender profiles of postgraduate students in medical specialties (registrars) from 1999 to 2006 and to identify factors affecting recruitment and retention of black and female trainees.

**Method:**

Review of faculty databases for race and gender data from 1999 to 2006. Distribution of an anonymous self-administered questionnaire to all registrars in 2005/2006.

**Results:**

The percentage of African registrars doubled from 10% to 19% from 1999 to beyond 2002. The percentages of Africans, Coloureds and Indians rose steadily from 26% to 46% from 1999 to 2005, as did that of women from 27% to 44%. The institution's perceived good reputation, being an alumnus and originating from Cape Town were common reasons for choosing UCT for training. A quarter of respondents reported knowledge of a friend who decided against studying at UCT for reasons which included anticipated racial discrimination. Black respondents (23%), particularly African (50%), were more likely to describe registrarship at UCT as unwelcoming than white respondents (12%). Specific instances of personal experience of discrimination were uncommon and not associated with respondents' race or gender. Registrars who had had a child during registrarship and those reporting discrimination were more likely to rate the learning and research environment as poor (Odds Ratio, 4.01; 95% CI 0.98 – 16.47 and 1.99 95% CI 0.57 – 6.97, respectively).

**Conclusion:**

The proportion of black and female registrars at the University of Cape Town has increased steadily from 1999 to 2006, most likely a result of systematic equity policies and procedures adopted in the faculty during this period. The data point to a need for policies to make the institution more welcoming to diversity and for strategies to address institutional culture and mentorship, with an aim to develop examples of best practices to share within and between institutions.

## Background

Institutions of Higher Education in the health sector in South Africa face a significant challenge in ensuring that graduates reflect the full racial [see Additional file 1] and gender diversity of the population [1-3]. In the health setting, apartheid policies, through the systematic exclusion of black students from both undergraduate and postgraduate training in health professions [4-6], left a deep shadow on human resource capacity for the country. For example, in 1967, the ratio of white doctors trained per million of the white population in South Africa was almost 100 times the equivalent ratio for Africans.[5] Although whites constituted less than 20% of the population, 83% of all doctors and 94% of all specialists in 1985 were white [7]. Since 1994, although admissions of black students to formerly predominantly white institutions increased substantially [8-10], the median percentage of African students studying medicine in 1999 remained approximately half that for whites [10].

Consequently, the profile of faculty members in training institutions for medicine, particularly at senior levels, remains dominated by white staff, particularly men. Moreover, selection processes to identify suitable candidates for specialist posts frequently encounter the apparent difficulty of finding suitably qualified and experienced black applicants. Further, while female students have for many years been the majority of undergraduate students around the country, they have been notably absent in the ranks of postgraduate medical specialists [9]. Evidence suggests that the experience of registrar training is qualitatively different for female postgraduates [11] and that gender discrimination experienced by women in specialist training is a major disincentive to specialization.

Whilst redress of racial and gender disparities in health science education are clearly linked to imperatives for social reconstruction in post-apartheid South Africa [6,12,13], there are also cogent educational reasons to seek a diverse student and staff population in higher education [14]. Increased student diversity during training has the potential to enhance students' cultural competence [15], facilitates the construction of identity and subsequent cognitive growth [16], promotes critical thinking and ability to understand diverse perspectives [17], is associated with improved academic performance, intellectual and social self-confidence, and satisfaction with the institution [18], may contribute to graduates' willingness to serve in under-resourced areas [19] and increases student motivation and preparedness for active citizenship in a democracy [14], all key challenges facing societies in transition, such as South Africa.

Moreover, improving the racial profile of health care providers in South Africa has also been suggested as likely to contribute to improved access to care and clinical outcomes [20]. For example, patients should be seen by health care providers who can speak their language and understand their cultural backgrounds, skills much easier acquired in learning contexts where both staff and students are reflective of the full diversity of a country's population. Thus, while concerns about racial and gender disadvantage in medical training are reported in many countries [20-28], it was the confluence of previous research at the University of Cape Town (UCT) into gender [29,30] and racial [30,31] discrimination with a national process of reconciliation [6,12] that prompted the UCT Health Sciences Faculty to pursue an institutional transformation process [32], driven not only by legal imperatives but also by a fundamental commitment to human rights.

Registrars, as postgraduate trainees in medical specialties are known in South Africa (or as residents in the US and Europe), are the cadre of human resources from which will develop the next generation of teaching staff. As a result, the Faculty's transformation process sought to focus on strategies for registrar recruitment using a systematic approach to increase the uptake of black and female trainees (Appendix 1) with a view to impacting on the staff profile in the institution in the long term. These strategies began to be developed in 1998 and addressed not only numeric targets but, particularly since 2003, institutional culture as a possible barrier, seeking to identify ways to make the learning, research and service environment more welcoming to registrars.

Registrar training involves a structured programme of clinical apprenticeship and formal learning which typically takes between 4 and 6 years to complete, depending on the discipline. During this period, they are registered as postgraduate students with the university, but work full-time delivering care in the health services, whilst gaining clinical skills under supervision of senior staff. In the Western Cape hospitals around Cape Town, there has been a systematic cut in budgets to central teaching hospitals over the past decade, which has put pressure on posts, teaching time and resources available for registrar training.

To evaluate the impact of strategies to enhance recruitment of black and female registrars, this study aimed to assess trends in the race and gender profile of registrars in the Faculty from 1999 to 2006, and to identify factors that facilitate or act as a barrier to recruitment and retention of black and female registrars.

## Methods

Faculty office records were reviewed for race and gender profile of all registrars who were registered for Master of Medicine degree for the period 1999 to 2006. In addition, a cross-sectional survey using an anonymous, self-administered and semi-structured questionnaire in English (see Additional file 2) was conducted with all registrars in the faculty in 2005/2006 (n = 469). The questionnaire explored demographics, including parental occupation, university of first graduation, factors influencing registrars' choice to study at UCT, knowledge of reasons given by registrars' friends or colleagues not to attend UCT (including others' anticipation of racial discrimination at UCT), experience of the recruitment process, personal experiences of discrimination and of the learning, teaching, service and research environment at UCT. Respondents were asked to rate the environment on a scale from 1 ('excellent') to 5 ('as bad as it can get') and comment on whether they found being a registrar at the UCT a welcoming experience. As pointed out earlier, race is used as a social construction in this paper, referring to apartheid categories African, Coloured, Indian and White, in order to reverse the consequences of past abuse under apartheid. The term 'black' is used to refer collectively to African, Coloured and Indian persons.

Piloting of the questionnaire was conducted with four registrars who were shortly to complete their training time in late 2004. Amendments to the questionnaire were made based on their feedback and on review by the Faculty Management responsible for postgraduate support.

Questionnaires were distributed in both hard copy and electronically by departmental and faculty structures for post-graduate support. The project was approved by the Faculty's Ethics Committee. [UCT REC Ethics Approval: # 154/2005]. Data were entered into an Excel spreadsheet and analysis were carried out using Stata version 9 (Stata Corporation, 2005).

## Results

**Figure 1:**
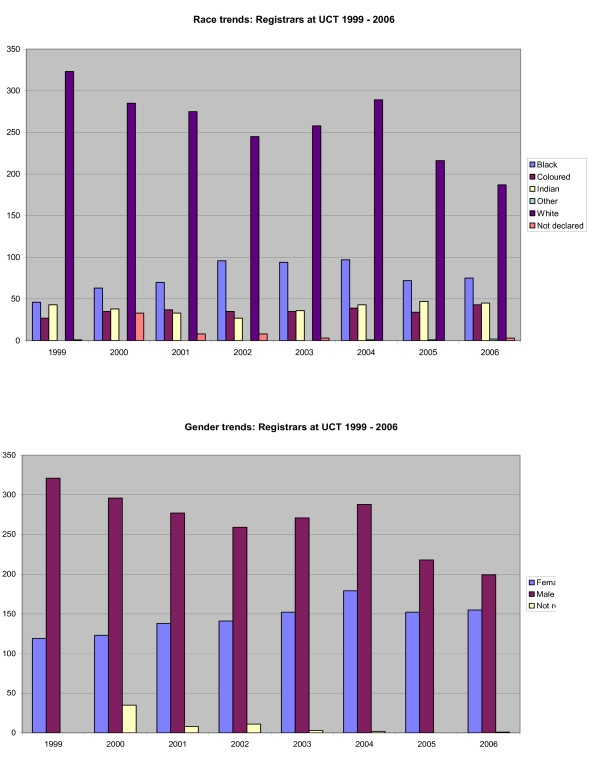
**Race and gender profile of registrars in the University of Cape Town Health Sciences Faculty, 1999–2006**.

During the period 1999 to 2006, the percentage of African registrars rose from 10% in 1999 to 19% in the period after 2002, while that of black registrars rose steadily from about 26% in 1999 to about 46% by 2006 (Figure 1). Similarly, the percentage of women rose from 27% to 44%. Race was not declared in 2% to 7% registrars during the period. Significant variation between disciplines was noted both for race and gender profiles. For example, in 2006, disciplines with the highest proportion of black registrars (> 50%) included Radiology, Public Health, Internal Medicine, Dermatology and Obstetrics and Gynaecology; those with the highest proportion of female registrars (>50%) included Public Health, Psychiatry, Paediatrics, Obstetrics and Gynaecology, Emergency Medicine, Clinical Pharmacology and Anaesthetics.

For the 2005/2006 questionnaire survey, despite repeated reminders, the response rate was 19% (n = 91). These respondents represented 22 out of 33 possible disciplines in the faculty. Higher response rates (> 33%) were present for registrars in Otorhinolaryngology, Psychiatry and Paediatrics as well as some smaller departments/disciplines such as Forensic Medicine and Virology.

The majority of respondents (80%) were between 25 and 34 years old (mean age 32.2 years (range 26 to 54) with no significant age difference between gender. Approximately two-thirds (65%) were male. The majority of respondents (90%) had registered at UCT within the preceding 5 years (since 2001). More than half (52%) were in either their first or second years of registrarship. The mean duration of registrar training at UCT was 2.6 years, (95% CI 2.1 – 3.1). Nineteen (21%) had a child before they started their registrarship and a further nine (10%) reported having had a child during their registrarship. Compared to the race and gender profile of all registrars in 2004 (Table 1), Africans were under-represented in the sample (7% versus 19% in the total registrar population; p = 0.003), whereas Indians were over-represented (21% versus 13%; p = 0.02). Six respondents (7%) declined to identify their race. The gender distribution of respondents was similar to the overall faculty totals.

**Table 1:** Comparison of race and gender of respondents and faculty, 2004 (percentages)

	African n = 6	Coloured n = 8	Indian n = 19	White n = 52	Did not declare n = 6	% of all respondents n = 91	% of all registrars in 2004
Female %	33	50	26	35	50	35	41
Male %	67	50	74	65	50	65	59
Total %	100	100	100	100	100	100	
% of all respondents	7	9	21	57	7	100	
% of all registrars in 2004	19	9	13	58	0		100

The majority of respondents were from three departments – Medicine (24%), Paediatrics (15%) and Psychiatry (14%). Most respondents (79%) were South African, whereas 18% were from other countries, particularly the United Kingdom. More than half (38/68) of the South African respondents were originally from the Western Cape Province and graduated at local universities (47% from UCT and 12% from the nearby University of Stellenbosch).

### Factors that influenced the registrars' choice of UCT

Only two registrars indicated that an alternative university had been their first choice, which meant we were unable to compare registrars by university of first choice. Reasons registrars gave for applying to UCT were primarily the perceived good reputation of the university or department (35%); their home being in Cape Town (24%); being a UCT alumnus (16%); and being attracted to Cape Town. No race or gender differences were found in reasons for choosing UCT, with the exception of female registrars who were more likely (41%) to cite reasons of Cape Town being their home compared to men (20%) (χ^2 ^= 4.00; p = 0.047). Of the 42 UCT alumni, 64% reported choosing UCT for either being an alumnus (n = 14) or because of the reputation of the University/department (n = 13), or both (n = 1).

Twenty-three respondents (25%) reported knowing a colleague who had chosen not to come to UCT for reasons that included feelings of anticipated racial or other discrimination (n = 8), family-related reasons (n = 4) and language (n = 2 – a lack of Afrikaans). More black (15%) and more African (50%) registrars reported anticipated racial discrimination as a reason for a colleague's decision not to come to UCT than for white registrars (2%) (Fishers exact tests p = 0.03 and p = 0.004, respectively).

### UCT as a welcoming environment

Fifteen percent reported finding UCT to be an unwelcoming environment. Black respondents (23%), particularly Africans (50%), were more likely to describe UCT as unwelcoming than white respondents (12%). Though the difference was not statistically significant for all black respondents (χ^2 ^= 1.52; p = 0.22), despite the small numbers, it was significantly higher for African registrars (Fishers exact test p = 0.02). Perceived unfriendliness (3 white registrars) and racial prejudice (2 black registrars) were cited as reasons. Respondents from the UK and Australia were least likely (8%) to report finding UCT unwelcoming, while South African registrars from outside the Western Cape were more likely (30%) to do so. A similar pattern was found amongst white registrars, with no UK-based registrars reporting feeling unwelcome, while 15% of Western Cape and 25% of non-Western Cape based registrars reported that UCT was unwelcoming.

### Registrar recruitment

Registrars reported commonly (44%) that they had learnt of the posts by word of mouth. However, the role of other staff in notifying registrars of posts was ubiquitous: 15% had learnt of a post from the Head of Department; 8% from other teaching staff; 9% from other registrars at UCT; and 10% from other colleagues. Only two registrars reported responding to an advertisement in the *South African Medical Journal *and one to a contact from the Faculty's Transformation Office. None of the six African registrars reported learning of the post from the Head of Department. No significant racial or gender differences were found in how registrars were notified of posts.

More than half the respondents (57%) were unaware of registrar entry requirements before applying and only one respondent indicated receiving the Faculty's brochure on registrar training prior to application, one of the existing recruitment strategies developed by the Transformation Portfolio (Appendix 1).

### Registrar experiences during the course of their training

Fourteen percent reported personally experiencing discrimination that had impacted on their training. Of these, half (n = 7) reported racial discrimination, one reported gender discrimination and five did not specify the type of discrimination. Both black and white registrars reported racial discrimination: Of the seven registrars citing racial discrimination incidents, three were white and 4 were black. There were no statistically significant differences in reported personal experience of discrimination by race or gender.

### Experiences of the Teaching, Learning, Research and Service environment

Two registrars, both females, reported part-time registrarship; Table 2 shows the ratings provided by respondents of their experiences of the Teaching, Learning, Research and Service environment at UCT. Registrars' ratings of the learning and teaching environments were significantly better than ratings of service and research environments (paired t-testing; teaching versus research p = 0.008; teaching versus service p = 0.004; learning versus research p = 0.03; learning versus service p = 0.03). The ranking of an environment as being 'excellent' or 'good' declined from close to 50% for teaching (47%) and learning (44%) to less than a third for research (32%) and service (26%). A sizeable proportion of registrars rated their learning (33%), teaching (27%), research (46%) and service (34%) environments as 'poor' or 'as bad as it can get.'

**Table 2:** Registrars' rating^# ^of the teaching, learning, research and service environment

Environment	n	Mean Score	SD	Median	% rating excellent or good
Teaching	89	2.7	1.0	3	47
Learning	88	2.8	1.1	3	44
Research	79	3.2	1.3	3	32
Service	89	3.1	1.0	3	26

^# ^Scores range from 1('excellent') to 5 ('as bad as it can get').

There were no statistically significant differences on bivariate comparisons by race, gender or having had a child for ratings in all four domains, although black registrars scored lower for teaching, research and service and white registrars lower for learning (Table 3). Registrars who reported having a child during their registrarship (7 men and 2 women) gave poorer ratings for the teaching environment compared to other registrars (3.1 versus 2.7), and they were four times more likely to rate the learning environment as 'poor' (OR = 4.01, 95% CI 0.98 – 16.47). There were non-significant poor ratings of the research environment in relation to registrars reporting personal experience of discrimination (OR = 1.99, 95% CI 0.57 – 6.97) and of the service environment in relation to registrars who did not report being attracted to UCT on the basis of being alumnus (OR = 2.42, 95% CI 0.62 – 9.50).

**Table 3:** Mean scores^# ^(SD) of respondents' rating of the teaching, learning, research and service environment

Predictor		Teaching	Learning	Research	Service
Race	White (n = 51)	2.7 (1.0)	2.9 (1.1)	3.1 (1.3)	3.1 (1.0)
	Black (n = 33)	2.8 (1.1)	2.8 (1.3)	3.4 (1.4)	3.3 (1.2)
Gender	Male (n = 58)	2.8 (1.0)	2.9 (1.0)	3.3 (1.4)	3.3 (1.0)
	Female (n = 31)	2.6 (1.1)	2.7 (1.3)	3.1 (1.1)	2.9 (1.0)
Children born during training	Yes (n = 9)	**3.1 (1.4)**	3.1 (1.4)	3.3 (1.3)	3.2 (1.0)
	No (n = 80)	**2.7 (1.0)**	2.8 (1.1)	2.9 (1.7)	3.0 (1.2)
Experience of UCT environment	a) Unwelcoming (n = 14)	2.8 (1.4)	2.9 (1.1)	3.2 (1.3)	3.2 (1.0)
	Welcoming (n = 72)	2.8 (1.0)	2.6 (1.4)	3.1 (1.5)	2.9 (1.1)
	b) Experienced discrimination (n = 13)	2.9 (1.3)	2.8 (1.1)	**3.7 (1.2)**	2.9 (1.0)
	No discrimination (n = 72)	2.7 (1.0)	2.8 (1.2)	**3.1 (1.3)**	3.2 (1.1)
	c) Experienced racial discrimination (n = 7)	**3.5 (1.1)**	3.0 (1.4)	**4.2 (0.8)**	3.3 (0.8)
	No racial discrimination (n = 78)	**2.6 (1.0)**	2.8 (1.1)	**3.1 (1.3)**	3.1 (1.1)
Reason for applying to UCT	a) High Reputation of UCT/department	2.7 (1.0)	2.7 (1.2)	3.2 (1.3)	3.2 (1.0)
	Did not cite reputation	2.7 (1.1)	2.9 (1.2)	3.2 (1.3)	3.1 (1.1)
	b) Cited being UCT alumnus	2.7 (1.1)	3.0 (1.2)	3.2 (1.2)	**2.8 (1.0)**
	Did not cite being alumnus	2.7 (1.1)	2.8 (1.2)	3.2 (1.3)	**3.2 (1.0)**

^# ^Scores range from 1('excellent') to 5 ('as bad as it can get').

Registrars who reported personal experience of racial discrimination (n = 7) gave a poorer assessment of the learning (Kruskal-Wallis testing; p < 0.05) and research (Kruskal-Wallis testing; p < 0.05) environments than those who did not report such discrimination (n = 78). Multivariate logistic regression analysis to identify predictors of poor environments (scores ≥ 3 versus ≤ 2) showed similar findings to those summarized in Table 3 for bivariate comparisons. For example, registrars with personal experience of racial discrimination were more likely to report the teaching environment as 'poor' (OR = 11.9; 95% CI 1.8–77.1) even when controlling for race. However, because of incomplete data for all the variables and modest fit, none of the models are presented here.

## Discussion

The gender and race distribution of respondents confirms the under-representation of women and black registrars at UCT. Changes documented in the gender and race profiles of registrars at UCT from 2001 to 2006 are modest but provide evidence of increasing recruitment of black and female registrars. Though not proven, these trends are most likely the result of systematic policies adopted by the Faculty to attract and retain talented black and female trainees [9,31,32]. These policies are similar in many respects to strategies considered and attempted at other formerly racially exclusive training institutions in South Africa to change their race and gender profile [33]. Given that modest shifts in profiles were achieved from a relatively low base, there is need for continued attention to ensure the implementation of strategies to make the institution environments welcoming for potential registrars, particularly those from disadvantaged groups. Ongoing attention to proactive affirmative action recruitment amongst undergraduates has also been noted in the US as a key strategy to address the health of minority groups [34].

As noted, [35,36] transformation is more than just about numeric targets. Unless the experience of studying or working at an institution is a positive one, attempts to recruit blacks and females will result in a 'revolving door' situation, where black/female staff members are not retained, or worse, feelings of alienation are exacerbated. About a third of the respondents in this study reported teaching and learning environments as 'poor'.' Although not as extreme as those reported by Winter and others [37], these findings are sobering, particularly given that registrars' reported ratings of learning and teaching that were better than those for service and research. The reasons for poorer ratings for service and research may lie in declining provincial health budgets in the Western Cape Province over the past 10 years, which have reduced funding available at tertiary level, where much of the training takes place. The budget cuts have led to a reduction in posts, an increased workload and reports that the 'hallways of most hospitals in this country echo with disillusioned, disgruntled, frustrated and fatigued voices overwhelmed by the demands of service delivery' [[38]: p63]. The importance of an appropriate balance between service demands and educational activities in resident training has been identified in other contexts [39]. Given increased service pressures, it is therefore usually research activity that is cut to the minimum [38] whereas teaching and learning, perceived as core functions for clinical teachers, are more likely to be protected [40].

Such a distinction may also reflect clinician-teachers' dedication to maintaining high levels of teaching in the face of an increasingly inhospitable service environment [40]. Indeed, the importance of clinical supervision, the extent and quality of teaching, and the availability of support for registrars' participation in conferences was noted to be an important predictor of South African registrars' willingness to persevere with training under stressful conditions [41]. The structured availability of learning opportunities was shown to be an important determinant of the quality of residency training in Family Medicine in the US [42]. Thus, even though the service environment may be pressurised by budgetary shortfalls, there are strategies to ensure that teaching and learning activities continue to meet registrars' academic and educational needs, that do not necessarily require additional resources. Such strategies include the use of shadowing, the allocation of protected teaching and learning time, and systematic mentorship by senior registrars and consultants, who, through a structured relationship, act as educational supervisors and guide registrars to access educational and research resources and networks. In the absence of such strategies, registrars' time risks being overwhelmed by service demands. Indeed, other studies have found that residents report that they have less time for teaching and research than they need or are satisfied with and spend more time than desirable on training in direct patient care [41,43].

In general, black registrars tended to rate teaching, research and service environments worse than white registrars, but the converse applied for the ratings of their learning environment, though these differences were not statistically significant. Although the numbers reporting personal experience of racial discrimination were low (n = 7), it was personal reporting of discrimination that was significantly associated with poorer assessments of learning and research environments. Such findings are in keeping with the international literature that illustrates the impact of gender [24,25,28,44-48] and race [21-23,27,49,50] discrimination on career development and with studies showing the ongoing salience of race in the undergraduate learning context in post-apartheid South Africa [51]. The impact on teaching and learning may be direct, in that the experience of discrimination may directly affect learning opportunities, or, alternatively, unrelated experiences at the same institution may flavour the nature of the learning experience for the trainee. The fact that white respondents were amongst those reporting personal experience of race discrimination points to the complexities of implementing Employment Equity policies that remain fair and non-discriminatory, a question raised in broader debates on post-apartheid transformation [52].

To what extent did UCT present a welcoming environment to potential trainees? Although there were no statistically significant differences by race in respondents' reporting knowledge of a colleague deciding not to apply to UCT (for any reason), there was a significant difference by race in relation to racial discrimination anticipated by colleagues, which was the most common single reason cited by respondents for others' decisions not to come to UCT (n = 7). Given that no differences were found in actual personal experience of racial discrimination reported by race amongst the same respondents, this most likely reflects an anticipation of institutional discrimination. Indeed, other studies have confirmed there is a real historical precedent for such perception [8,31] even if practices have changed in recent times. Further, given the broader patterns of racial stratification in South Africa, still persistent post-apartheid, this result is also a function of registrar applicants' life experiences, in that black applicants are more likely to have black friends who, in turn are more likely to experience discrimination than white colleagues or white friends of white colleagues. As a result, it is not surprising that black respondents, particularly African respondents, were more likely to describe UCT as unwelcoming than white respondents. Measures to address recruitment and retention also therefore need to address the elements of an institutional culture that contribute to making the institution a welcoming environment for all [53].

This insider-outside dynamic, however, was not solely based on race. Over a third of respondents reported being alumni of UCT and returning 'home' was an important factor that determined registrars' choice to study at UCT. Hence, being an outsider to both the institution and the city may cause discomfort and anticipation of discrimination. Even among white respondents, registrars who were not originally from Cape Town were about 66% more likely to find UCT unwelcoming than their white counterparts who were from Cape Town. Indeed, it was striking that the registrars who were least likely to describe UCT as unwelcoming were those from outside South Africa, even more so than white registrars from South Africa. Such differences may partly relate to the experience of the culture of Cape Town and not only that of the institution, and confirm the importance of orientation and induction as important strategies for transformation, as much as re-orienting how staff members are made to feel welcome in the course of their attachment.

Although women have outnumbered male medical students at undergraduate level for a number of years at UCT, they still constitute a minority in postgraduate programmes [8]. Women, in particular; carry a double load of parenting whilst undergoing specialist training [24,44,48,54]. Saloojee and Rothberg [11] highlighted the potential for gender discrimination experienced by women in registrar training in paediatrics in South Africa. Findings in this study confirm that having a child during registrarship was significantly associated with poorer rating of the teaching environment. Scores were also worse for learning, research and service, though differences for gender were not statistically significant.

Notably, female gender alone was not predictive of differences in ratings; indeed, women tended to rate their experiences in all four domains better than did men, though not significantly so. This is not to say that gender discrimination is not an issue for improving the quality of registrar training. At least one respondent cited an instance of gender discrimination in her registrar experience, and it is clear in Figure 1 that there is a gender disproportion by department in the faculty. However, it appears that it is the challenge of having a child during training that is the most important determinant of the learning experience, which points to the importance of institutional support in the form of job-sharing or other forms of flexible work arrangements that can accommodate parenting responsibilities [11,54]. In other contexts, flexible working arrangements can be as common as comprising 20% of posts in paediatrics and psychiatry [55]. Such strategies should apply for both women and men, both in the interests of achieving gender equality and because of evidence that the stress of caring for a child whilst undergoing residency training may be more stressful for men than for women residents [56].

Of note, as well, is the strong evidence that UCT continues to draw most of its registrar pool from local graduates. This trend has both positive and negative implications. As a way to groom talented students, recruiting one's own alumni offers an opportunity to mentor students from under-graduate years, and points to the importance of senior staff role models who actively encourage students to return for registrar training. The role of the Head of Department (HoDs) is thus critical in this process and HoDs were the most common single source of information about posts other than word of mouth. However, it also means that the institution is perhaps not making optimal use of the widest pool of potential applicants, a particular concern given perceptions of UCT being unwelcoming to applicants. Registrars who did not apply to UCT on the basis of being an alumnus were more than twice as likely to rate the service environment poorly, probably reflecting their lack of familiarity with the difficulties of working in a province where budgetary constraints impact the service platform.

There were many limitations to this study. First, a response rate of 19% means the sample is not representative of the registrar population at UCT, particularly given the over-representation of Indian and under-representation of African registrars amongst the responders compared to the total registrar population. However, included in the respondents were 2/3 of all clinical disciplines and the only large clinical absent amongst responders was the Department of General Surgery. Further, the age range of responding registrars is broadly similar to that anticipated for registrars in the faculty. The low response rate is also not unusual for postal questionnaires and is, in fact, slightly higher than the response to an Institutional Climate survey held at UCT in 2007 [57]. However, studies amongst equivalent medical trainee populations have achieved much higher response rates, to the order of 50 to 80%,[37,41,47,56,58] albeit using different methodologies.

Respondents in this survey may therefore have been systematically different to non-responders, particularly if they had personal experience of discrimination or felt aggrieved by adverse experiences which they wished to voice. Further, responses to the questions may have been less than entirely honest, despite assured anonymity of the questionnaire. The direction of the response bias, if any, may have been either to under- or over-state problems. We can also not discount recall bias affecting the study, since respondents were asked to recall experiences or incidents over periods that may have been up to four years prior to interview. In such cases, it would be likely to underestimate the prevalence of reported racial or gender discrimination. Further, the small sample size may have made it impossible to distinguish large differences in practice and policy between and even within departments, a point raised in feedback discussions with registrars after the study.

Nonetheless, despite the room for selection and measurement error, the findings from this study appear consistent with results from other research in the faculty investigating questions of race, gender and transformation [30,51,59] and from feedback discussions with registrars following the study. The percentage of respondents reporting personal experiences of discrimination was similar to that found in the UCT climate surveys of staff across faculties [57,60]. Inasmuch as they point to issues which an institution of higher learning might consider as strategic challenges for transformation, they provide pointers to further action needed, as well as potential baseline data for evaluating the future effectiveness of such strategies. The presentation of the findings to registrars has also spurred interest from the registrars to conduct their own research and to initiate more regular faculty-wide monitoring of the registrar programme.

## Conclusion

We believe the study provide tentative evidence to support the following conclusions: In the face of relatively high levels of dissatisfaction with the experience of research and service in this study, registrars still valued the reputation of UCT and their department highly as centres of excellence. Recruitment to registrar posts occurs predominantly through word of mouth, and departmental heads play a critical role in facilitating informal networks of recruitment. Increasing the diversity of the pool of suitable candidates for selection into registrar programmes is therefore a key leadership challenge. Personal experiences of racial and gender discrimination, whilst relatively uncommon at UCT, were reported and must act as signals to address the institutional culture of the organization, particularly given the evidence presented regarding a fairly high level of feeling unwelcome at the institution, especially for South African registrars whose home is not in the Western Cape. These provisional findings, which should ideally be confirmed in further research with higher response rates, coupled with subsequent interventions should aim to develop best practices to share within and between institutions, if South Africa's health sector human resource needs are to be met.

## Competing interests

LL was the Portfolio holder for Transformation and Equity and SK was the Portfolio holder for Postgraduate Support, both faculty management positions in the Health Sciences Faculty at the University of Cape Town in South Africa at the time this research was conducted. The research implicitly evaluates some of the interventions developed by Faculty management to address issues of race and gender diversity in the Faculty. A conflict of interest may be perceived in relation to seeking outcomes favourable to the faculty. However, the study was conducted without preconceived expectations and student BX was employed to conduct the research, independent of any direct control from faculty management. As evident in the final report, reflected are both successes and failure of the faculty's interventions, presented as fairly and dispassionately as possible in the paper.

## Authors' contributions

LL conceptualised the study, led the drafting of the protocol and measurement instruments, conducted some of the analyses and write up. SK contributed to the development of the study conceptualisation, protocol and instruments, and contributed to the write up of the study. BX assisted in the development of the protocol and measurement instruments, conducted the data collection and primary analysis, undertook the first draft of the paper and commented on subsequent drafts. All authors read and approved the final manuscript

## Appendix 1

Employment Equity measures for registrar recruitment

• Sign-off on all departmental recommendations for registrar appointments based on equity targets

• Equity representatives on selection committees for registrars

• Clear equity requirements included in registrar advertisements

• Wide distribution of adverts to networks likely to recruit persons from designated groups

• Proactive information provision to senior medical students about career options and requirements needed for specialization at UCT

• Development and annual update of a database of black UCT medical graduates encouraged to return to UCT to specialize

• Heads of Departments asked to mentor students from designated group to encourage return for registrarship

• UCT funding for bridging posts to enable applicants to gain experience or qualifications to enter the registrar programme

• Harmonisation of Employment Equities policies and procedures with the Provincial Health Department

• Institutional culture intervention to address barriers to potential registrars taking up posts

## Pre-publication history

The pre-publication history for this paper can be accessed here:



## Supplementary Material

Additional file 1 **Note on use of race terminology**. Explanation on the use of racial terminology in the paper. Click here for file

Additional file 2 **Final questionnaire**. Questionnaire used in the study. Click here for file
