# Supplementary material for: A survey of trainee specialists experiences at the University of Cape Town (UCT): Impacts of race and gender
Source: BMC Med Educ. 2009 May 27;9:26. doi: 10.1186/1472-6920-9-26 (PMC2696443; doi:10.1186/1472-6920-9-26)
Supplement: Additional file 1 — Note on use of race terminology. Explanation on the use of racial terminology in the paper. [file 1472-6920-9-26-S1.doc]

**Note on use of race terminology:**

The terms African, Coloured, Indian and White are used purposively in this study as part of a current attempt to redress past racial inequalities, recognizing that these racial descriptors were used to foster discrimination under apartheid and widely abused in biomedical research. Their use in this study is not intended to legitimize such distinctions other than as social constructions. The term ‘black’ is used to refer collectively to African, Coloured and Indian persons.
