# Supplementary material for: A survey of trainee specialists experiences at the University of Cape Town (UCT): Impacts of race and gender
Source: BMC Med Educ. 2009 May 27;9:26. doi: 10.1186/1472-6920-9-26 (PMC2696443; doi:10.1186/1472-6920-9-26)
Supplement: Additional file 2 — Final questionnaire. Questionnaire used in the study. [file 1472-6920-9-26-S2.doc]

Date: 12/4/2005

Dear Participant:

The UCT HSF is doing a study of to evaluate the process of recruitment and induction of its registrars and whether interventions to promote diversity in the recruitment of registrars have had any impact. This will serve as baseline for future evaluation.

We urge you to assist by completing the questionnaire that accompanies this letter.

If you do not wish to participate, you will not be penalised in any way. You are free to decline to participate. However, we would like to encourage you to help us identify potential obstacles to making the experience of training as a registrar at UCT a positive experience, so that we can improve the programme and the experience.

The information submitted by you will be completely anonymous and will not be able to be traced to any individual.

However, if you are willing to be interviewed about your experiences, we have put a section into the questionnaire where you can indicate how you can be contacted for an interview. We will keep any interview information confidential and in the reports, no data will be presented that can be linked to any individual.

The report will form part of a current NRF-funded research project and we will arrange both a written and verbal report (with opportunity for questions) for registrars. Once the report is finalized, it will be submitted to the Faculty and posted on the Transformation Website, should you want to access it.

For further information or queries please contact Bonga Xaba at the e-mail address below. Your participation will be greatly appreciated.

Thanking you in advance.

Bonga Xaba ([xbxbon004@mail.uct.ac.za](mailto:xbxbon004@mail.uct.ac.za))

Assisted by Joanne Stevens ([jstevens@cormack.uct.ac.za](mailto:jstevens@cormack.uct.ac.za))

Faculty Transformation and Equity portfolio,

Dean’s Office,

Health Sciences Faculty, Barnard Fuller Building

**UCT REC Ethics Approval: # 154/2005**

7.2 **QUESTIONNAIRE**

*Thank you for taking the time to fill out this questionnaire. Please circle/tick the option that applies to you where appropriate. Some questions require a brief written response, please answer them as honestly as you can.*

| 1. AGE: (years) |  | | |  | | | 2. SEX: | | | | | | | | | | | M | F |
| --- | --- | --- | --- | --- | --- | --- | --- | --- | --- | --- | --- | --- | --- | --- | --- | --- | --- | --- | --- |
| 3. RACE | Black | White | | | | Coloured | | Indian | | | | Other | | |  | | | | |
| 4. Do you have a disability? | | | | | | | Y | | | | N | | |  | | | | | |
| If yes, what is your disability? | | | | | | |  | | | | | | | | |  | | | |
| 5. Do you have a child/children? | | | | | | | Y | | | | N | | |  | | | | | |
| 5.1 Did you have a child/children when you started your registrarship at UCT? | | | | | | | Y | | | | N | | |  | | | | | |
| 6. Where were you living before coming to UCT as a registrar? | | | | | Country: | | | | | | | | Province: | | | | | | |
| 7. When did you obtain your MBCHB or the equivalent thereof? | | | | | | | | | | | | | | | |  | | | |
| 7.1 From which university? | | | | | |  | | | | | | | | | | | | | |
| 8. When was your first year of registrarship? | | | | | | | | |  | | | | | | | | | | |
| 8.1 In which Department? | | |  | | | | | | |  | | | | | | | | | |
| 9. Do you have part-time registrarship? | | | | | | | Y | | | | N | | |  | | | | | |
| 10. Have you found being a registrar at UCT a welcoming experience? | | | | | | | | | | | | | | | | | Y | N |  |
| 10.1 If not, why | | | | | | | | | | | | | | | | | | | |
|  |  | | | | | |  | | | | | | | | |  | | | |
|  |  | | | | | |  | | | | | | | | |  | | | |

| [We would like to interview registrars who have had difficulties adapting or moving to Cape Town or who found Cape Town / UCT an unwelcoming place. To do this, we need to know who to contact. If you are willing to be interviewed about your experience, please place you name and contact details (phone or email) here:  Name (if willing to be interviewed) ___________________________  Phone: email: |
| --- |

| 11. Do you know of friends who chose not to come to UCT? | | | | | Y | | N | |  | | |
| --- | --- | --- | --- | --- | --- | --- | --- | --- | --- | --- | --- |
| 11.1 If yes, what were their reasons? | | |  | | | | | | | | |
| 12. Which university was your first preference? | | | |  | | | | | | | |
| 13. What was the reason for your first preference? | | | | |  | | | | | | |
| 14. How did you learn about the registrar post you now occupy? (Tick as many as apply.) | | | | | | | | | | | |
|  | SAMJ advert |  | Encouraged by head of department | | | | | | |  |  |
|  | Word of mouth |  | Encouraged by another registrar | | | | | | |  |  |
|  | Contacted by UCT transformation office |  | Encouraged by some other colleague | | | | | | |  |  |
|  | Encouraged by UCT member of staff |  | Other source (specify): | | | | | | |  |  |
| 15. When you applied, were you aware of entry requirements for registration training? | | | | | Y | N | |  | | | |
| 15.1 If yes, where did you get the information? | | | | | | | | | | | |
| 16. Have you experienced any discrimination at UCT since you enrolled with UCT? | | | | | Y | N | |  | | | |
| 16.1 If yes, explain | | | | | | | | | | | |

| 17. How would you rate your experience of the academic environment from 1(excellent) to 5 (as bad as it can get) N/A = Not applicable  Please circle the code that BEST describes your experience  Quality of teaching you receive 1 2 3 4 5 N/A |
| --- |
| Quality of your learning experience 1 2 3 4 5 N/A |
| Quality of your research experience 1 2 3 4 5 N/A |
| Quality of your service environment 1 2 3 4 5 N/A |

**Thank you for your time - Your efforts are greatly appreciated!!!**

Please return this form to Mr Bonga Xaba, c/o the Transformation and Equity portfolio, Dean’s Office, Health Sciences Faculty, Barnard Fuller Building by 27^th^ May 2005. You can do so by post, or by email (to [xbxbon004@mail.uct.ac.za](mailto:xbxbon004@mail.uct.ac.za) or to [jstevens@cormack.uct.ac.za](mailto:jstevens@cormack.uct.ac.za)) if you are happy with direct email communication.
